# Supplementary material for: DMRforPairs: identifying Differentially Methylated Regions between unique samples using array based methylation profiles
Source: BMC Bioinformatics. 2014 May 15;15:141. doi: 10.1186/1471-2105-15-141 (PMC4046028; doi:10.1186/1471-2105-15-141)
Supplement: Additional file 2 — DMRforPairs output for the comparison of A431-MCF7 and NA17018-NA17105. Please start from the HTML files in each folder. Available via the BMC Bioinformatics website. [file 1471-2105-15-141-S2.zip › 1394847754114233_MOESM2_ESM/A431_MCF7/figures/12176.pdf]

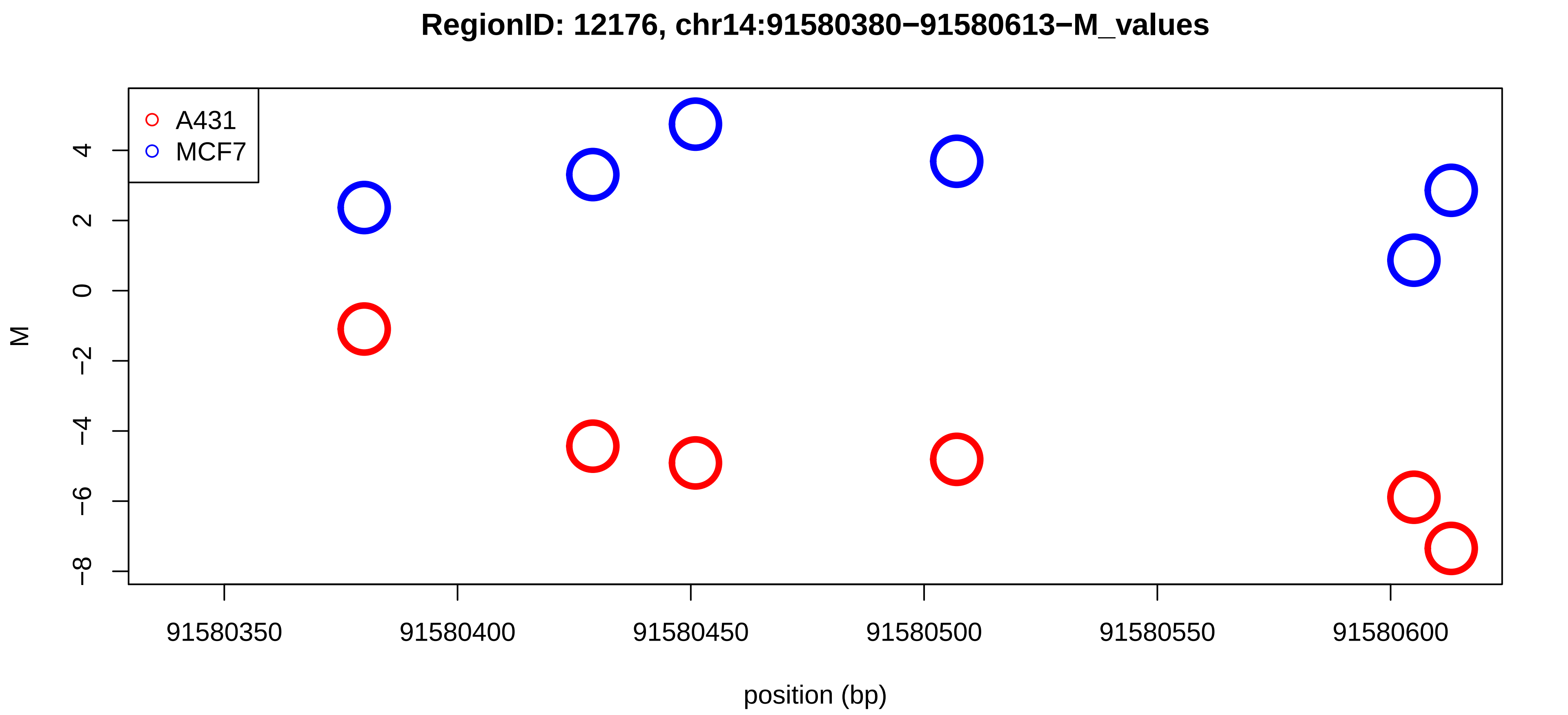

RegionID: 12176, chr14:91580380−91580613−M_values
A431
MCF7
M
4
2
0
−2
−4
−6
−8
91580350
91580400
91580450
91580500
91580550
91580600
position (bp)

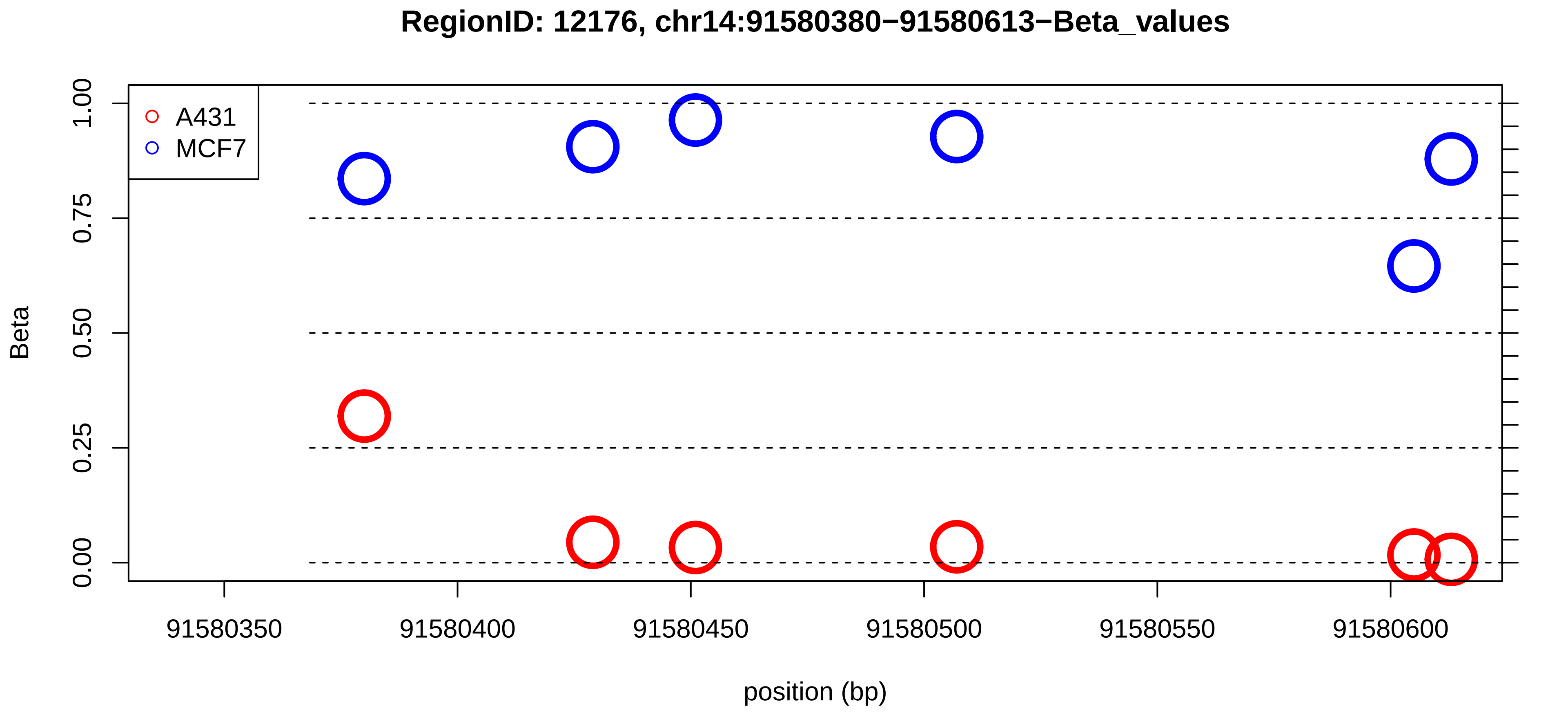

RegionID: 12176, chr14:91580380−91580613−Beta_values
A431
MCF7
Beta
1.00
0.75
0.50
0.25
0.00
91580350
91580400
91580450
91580500
91580550
91580600
position (bp)

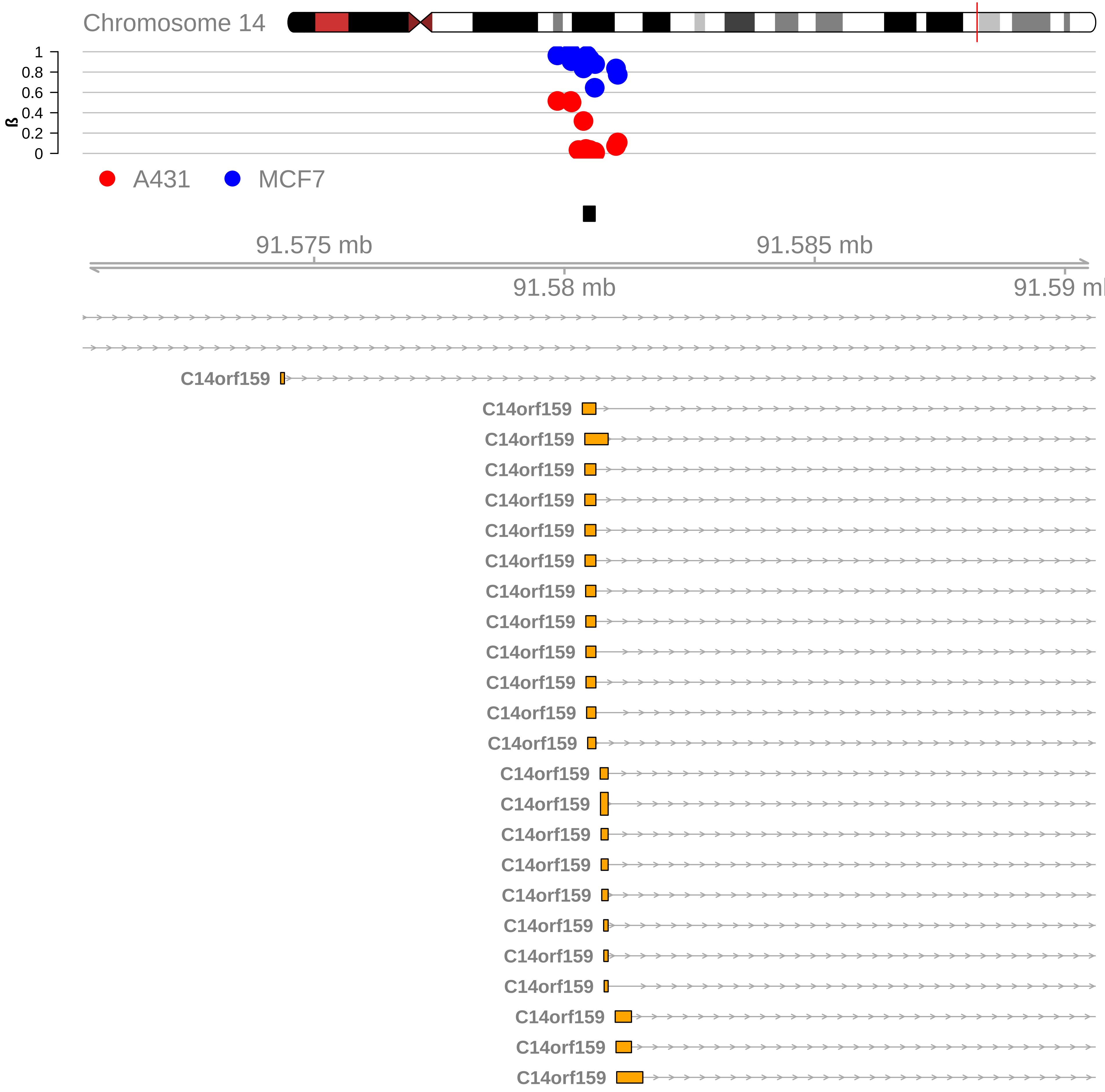
Chromosome 14
ß
1
0.8
0.6
0.4
0.2
0
A431
MCF7
91.575 mb
91.585 mb
91.58 mb
91.59 mb
C14orf159
C14orf159
C14orf159
C14orf159
C14orf159
C14orf159
C14orf159
C14orf159
C14orf159
C14orf159
C14orf159
C14orf159
C14orf159
C14orf159
C14orf159
C14orf159
C14orf159
C14orf159
C14orf159
C14orf159
C14orf159
C14orf159
C14orf159
C14orf159
